# Differential splicing choices made by neurons and astrocytes and their importance when investigating signal-dependent alternative splicing in neural cells

**DOI:** 10.3389/fnmol.2023.1214439

**Published:** 2023-07-03

**Authors:** Paul S. Baxter, Owen Dando, Giles E. Hardingham

**Affiliations:** ^1^Edinburgh Medical School, UK Dementia Research Institute, The University of Edinburgh, Edinburgh, United Kingdom; ^2^Centre for Discovery Brain Sciences, Edinburgh Medical School, University of Edinburgh, Edinburgh, United Kingdom

**Keywords:** neurexin, alternative splicing, epigenetics, excitotoxicity, astrocyte-specificity

## Abstract

A variety of proteins can be encoded by a single gene via the differential splicing of exons. In neurons this form of alternative splicing can be controlled by activity-dependent calcium signaling, leading to the properties of proteins being altered, including ion channels, neurotransmitter receptors and synaptic cell adhesion molecules. The pre-synaptic cell adhesion molecule Neurexin 1 (*Nrxn1*) is alternatively spliced at splice-site 4 (SS4) which governs exon 22 inclusion (SS4^+^) and consequently postsynaptic NMDA receptor responses. Nrxn1 was reported to be subject to a delayed-onset shift in *Nrxn1* SS4 splicing resulting in increased exon 22 inclusion, involving epigenetic mechanisms which, if disrupted, reduce memory stability. Exon inclusion at SS4 represented one of hundreds of exons reported to be subject to a genome-wide shift in fractional exon inclusion following membrane depolarization with high extracellular K^+^ that was delayed in onset. We report that high K^+^ does not increase the SS4^+^/SS4^−^ ratio in cortical neurons, but does induce a delayed-onset NMDA receptor-dependent neuronal death. In mixed neuronal/astrocyte cultures this neuronal death results in an increase in the astrocyte: neuron ratio, and a misleading increase in SS4^+^/SS4^−^ ratio attributable to astrocytes having a far higher SS4^+^/SS4^−^ ratio than neurons, rather than any change in the neurons themselves. We reassessed the previously reported genome-wide delayed-onset shift in fractional exon inclusion after high K^+^ exposure. This revealed that the reported changes correlated strongly with differences in exon inclusion level between astrocytes and neurons, and was accompanied by a strong decrease in the ratio of neuron-specific: astrocyte-specific gene expression. As such, these changes can be explained by the neurotoxic nature of the stimulation paradigm, underlining the importance of NMDA receptor blockade when using high K^+^ depolarizing stimuli.

## Introduction

In mammalian neurons, electrical activity induces changes in gene expression with a variety of long-term consequences, including alterations in survival, metabolism, development, and synaptic strength/connectivity (Bell and Hardingham, 2011; Hardingham et al., 2018; Lee and Fields, 2021). Many of these changes involve the up- or down-regulation of transcription via the alteration of Ca^2+^-responsive transcription factors that bind to the promoters of activity-dependent genes. Additionally many genes exhibit activity-dependent alternative splicing, whereby exon splicing efficiency is controlled by activity-dependent signals acting on specific RNA binding proteins (Furlanis and Scheiffele, 2018; Jacko et al., 2018; Farini et al., 2020). The effects of activity-dependent alternative splicing are wide-ranging, including synaptic and neuronal development, specification, and activity (Furlanis and Scheiffele, 2018). Consistent with its role in development and synapse biology, misregulation of activity-dependent alternative splicing has been proposed to contribute to autism spectrum disorder (ASD) phenotypes (Parikshak et al., 2016; Quesnel-Vallières et al., 2016; Quesnel-Vallieres et al., 2019).

The Neurexins (Nrxn1-3) are cell adhesion molecules that play key roles in synapse development and in defining synaptic properties (Sudhof, 2017, 2018), and Neurexin mutations have been implicated in ASD and other neuropsychiatric disorders (Tromp et al., 2021). Nrxn genes exhibit extensive alternative splicing, the functional consequences of which have been extensively studied (Sudhof, 2017), particularly at alternative splice site 4 (SS4), which determines the inclusion (SS4^+^) or exclusion (SS4^−^) of a 90 nt exon. Alternative splicing at SS4 determines Nrxn affinity for neuroligins as well as specifying other binding partners including cerebellins and leucine-rich repeat transmembrane proteins (Sudhof, 2017, 2018; Yuzaki, 2018), and also determines the impact of neurexins on post-synaptic NMDA and AMPA receptor mediated responses (Dai et al., 2019).

Given the profound functional impact of SS4^+^ vs. SS4^–^ identity, the potential for SS4 splicing to be regulated is of considerable interest. Strikingly, Ding et al. (2017) made the case that neuronal activity triggers an increase in SS4^+^/SS4^−^ ratio. They also report a series of other activity-dependent alternative splicing events obtained from genome-wide analysis of RNA-seq data. A key activity-inducing paradigm employed to show increased SS4^+^/SS4^−^ ratio was a 10 min exposure of cortical neuronal cultures to 50 mM KCl, to induce membrane depolarization (Ding et al., 2017). This resulted in a delayed (peaking 24 h post-stimulation) increase in the ratio of the SS4^+^:SS4^−^ ratio (Ding et al., 2017). However, strong KCl-induced depolarization can trigger excitotoxic neuronal death due to inappropriate activation of NMDA receptors (NMDARs) (Schramm et al., 1990; Ramnath et al., 1992), requiring the use of NMDAR antagonists to prevent toxicity in order to study KCl-induced Ca^2+^ influx through voltage-gated Ca^2+^ channels (Xia et al., 1996; Zhou et al., 2009; Qiu et al., 2016). Depolarisation of neurons removess the voltage-dependent blockade of NMDA receptors by Mg^2+^ which, coupled with an elevation of ambient glutamate due to depolarisation-induced vesicular release and impaired glial cell uptake, can lead to chronic NMDAR activity, including of extrasynaptic NMDARs which are preferentially coupled to pro-death cascades (Soriano and Hardingham, 2007; Wahl et al., 2009; Parsons and Raymond, 2014).

Since NMDAR antagonists were not employed by Ding et al., we investigated whether their stimulation paradigm is neurotoxic, potentially confounding their conclusions regarding Nrxn1 alternative splicing and indeed the hundreds of other genome-wide events reported. We show here that these events, including exon inclusion at SS4, are likely to be an artefact of the stimulation conditions, which lead to a death of neurons, and a subsequent enrichment of the remaining astrocytes in the culture, which make different splicing choices to neurons, strongly favouring exon 22 inclusion (SS4^+^). Our conclusions align well with a study that also investigated this issue while our own study was in progress (Liakath-Ali and Sudhof, 2021).

## Results and discussion

**Figure 1:**
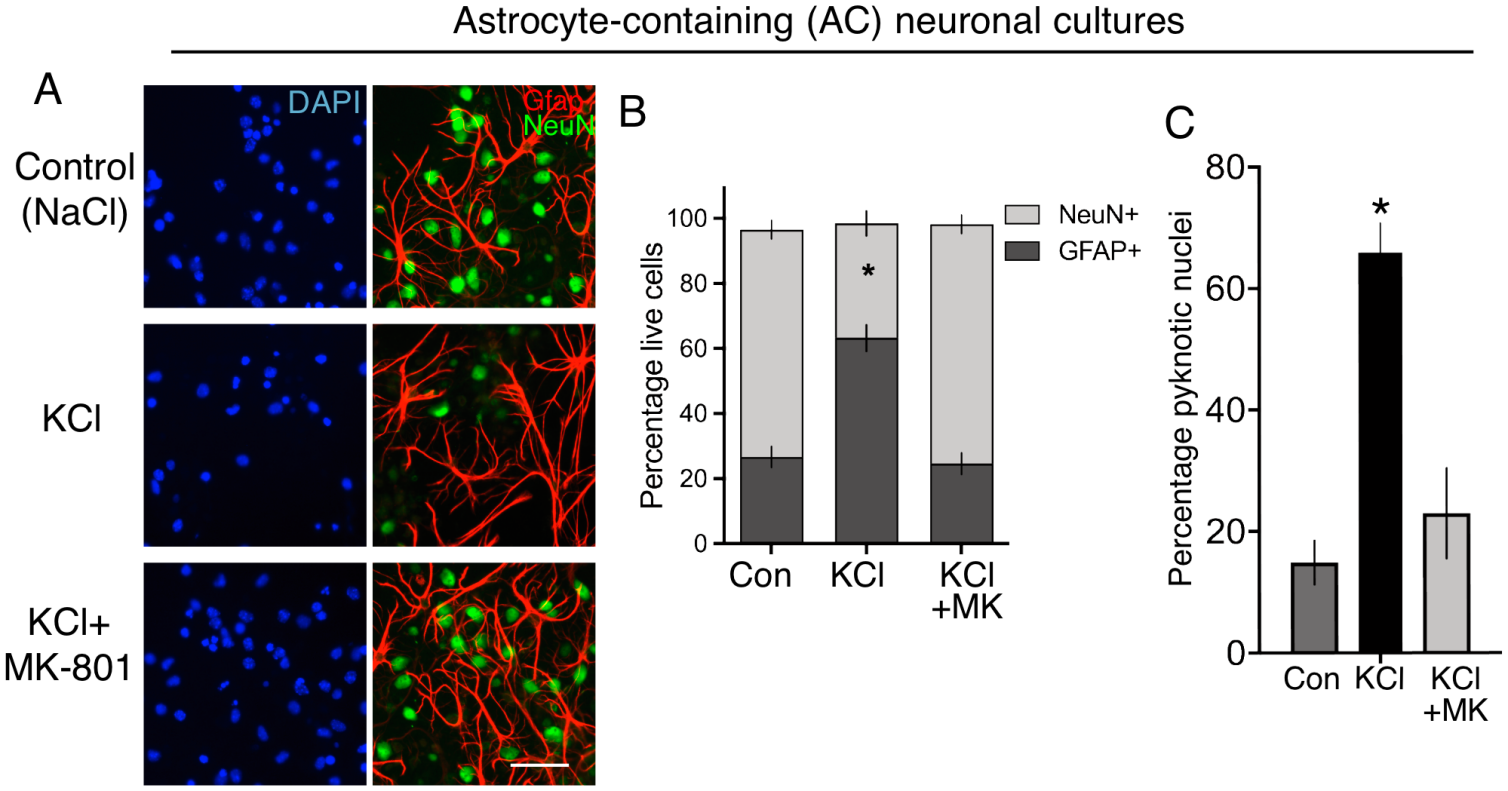
**(A)** Immunofluorescent labelling of Div 12 cortical cultures prepared as per Ding et al., 24 h after the indicated treatments, applied for 10 min. DAPI was used as a pan-nuclear stain, with antibodies to NeuN and Gfap employed to identify neurons and astrocytes, respectively. Scale bar:25 μm. **(B)** Quantification of the images shown in **(A)**. One way Anova: *F* (1.016, 3.049) = 66.01, *p* = 0.0037, *n* = 4. * denotes Tukey’s post-hoc comparison of Con (NaCl) vs. KCl, *p* = 0.0066. For each condition, 691–466 Gfap positive and 3,932–516 NeuN-positive cells were analysed in total across 4 independent experiments. **(C)** Quantification of cell death of cortical cultures prepared as per Ding et al., 24 h after the indicated treatments, applied for 10 min. One way Anova: *F* (2, 9) = 24.39, *p* = 0.0002, *n* = 4. * denotes Tukey’s post-hoc comparison of Con (NaCl) vs. KCl, *p* = 0.0003. For each condition, 6,752–3,545 cells were analysed were analysed in total across 4 independent experiments.

**Figure 2:**
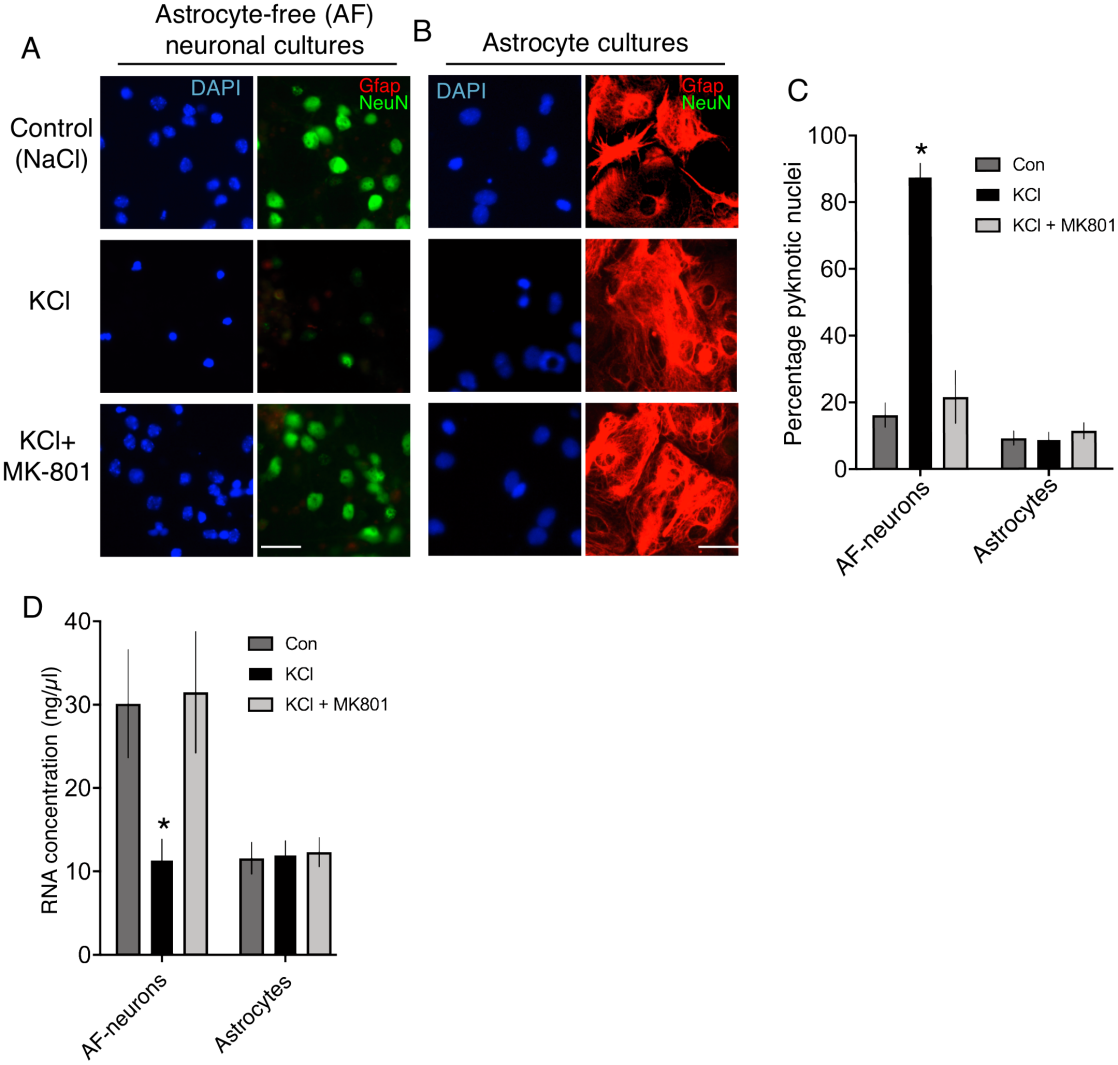
**(A,B)** Immunofluorescent labelling of astrocyte-free cortical neuronal cultures **(A)** and neuron-free astrocyte cultures **(B)** 24 h after the indicated treatments, applied for 10 min. DAPI was used as a pan-nuclear stain, with antibodies to NeuN and Gfap employed to identify neurons and astrocytes, respectively. Scale bar: 25 μm. **(C)** Quantification of cell death of cultures described in **(A)** and **(B)**. One way Anova for astrocyte-free neuronal culture data: F (2, 9) = 51.112, *p* < 0.0001, *n* = 4. * denotes Tukey’s post-hoc comparison of Con (NaCl) vs. KCl, *p* < 0.0001. One way Anova for astrocyte culture data: F (2, 9) = 0.4192, *p* = 0.6698, *n* = 4. For each condition, 3,175–3,001 cells from neuron-free-astrocyte cultures and 5,528–3,245 cells from astrocyte-free (AF)-neuronal cultures were analysed in total across 4 independent experiments. **(D)** RNA was extracted from cultures treated as per **(C)** and quantified. One way repeated measures Anova for astrocyte-free neuronal culture data: *F* (1.862, 16.76) = 11.99, *p* = 0.0007, *n* = 10. * denotes Tukey’s post-hoc comparison of Con (NaCl) vs. KCl, *p* = 0.0052. One way repeated measures Anova for astrocyte culture data: *F* (1.894, 17.04) = 0.5324, *p* = 0.5324, *n* = 10.

We created standard cortical neuronal cultures from embryonic mouse cultures in Neurobasal/B-27, similar to Ding et al. and generated a mixed population of 85% NeuN-positive neurons, 15% GFAP-positive astrocytes (astrocyte-containing (AC)-neuronal cultures, Figures 1A,B). Performming the KCl stimulation (10 min, no NMDA receptor antagonist) performed by Ding et al. led to substantial neuronal death 24 h later (Figures 1A,C), attenuated by the NMDA receptor antagonist MK-801 [Figure 1C, consistent with previous studies (Schramm et al., 1990; Ramnath et al., 1992)]. As a result, the proportion of astrocytes (GFAP-positive cells) in the culture post-KCl stimulation was much higher than control (NaCl) or KCl + MK-801 conditions (Figure 1B), since astrocyte viability is not affected by membrane depolarization. To study neurons and astrocytes in isolation we created astrocyte-free neuronal cultures (AF-neuronal cultures) by treating neuronal cultures with an anti-mitotic (AraC) on the day of plate-down, which limits astrocyte contamination to <0.1% (Hasel et al., 2017). We also employed neuron-free astrocyte cultures as a comparison (Baxter et al., 2015). 10 min KCl stimulation of AF-neuronal cultures resulted in NMDAR-dependent neuronal death 24 h later (Figures 2A,C). KCl did not trigger death in astrocyte cultures (Figures 2B,C). We also isolated RNA from cells exposed to these conditions and, consistent with KCl neurotoxicity, recovered less RNA in the AF-neuronal cultures treated with KCl, an effect rescued by MK-801 (Figure 2D). RNA amounts recovered from the astrocytes were unaffected by KCl stimulation.

**Figure 3:**
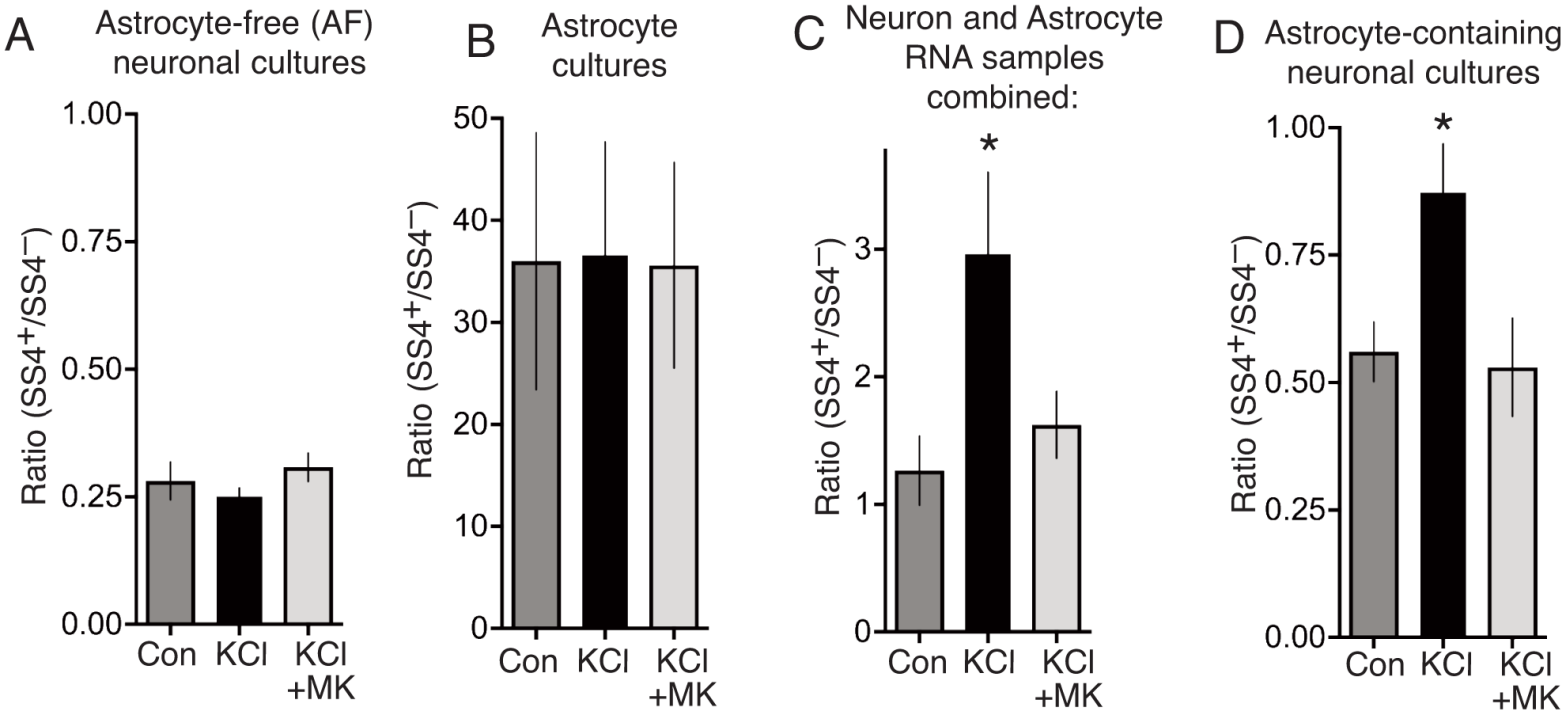
**(A)** Astrocyte-free cortical neuronal cultures were stimulated as indicated, for 10 min, RNA extracted 24 h later, and SS4^+^/SS4^−^ ratio calculated by qPCR using primer pairs specific for SS4^+^ and SS4^−^ isoforms of *Nrxn1*. One way Anova: *F* (1.271, 7.626) = 3.271, *p* = 0.21055, *n* = 7. **(B)** Neuron-free astrocyte cultures were treated and processed as per **(D)**. One way Anova: *F* (2, 17) = 0.0017, *p* = 0.998, *n* = 7. **(C)** Equal culture areas of astrocyte-free cortical neuronal cultures and neuron-free astrocyte cultures were treated as indicated, and their RNA extracted 24 h later. The RNA samples were then mixed for each stimulation, and the net SS4^+^/SS4^−^calculated. One way Anova: *F* (1.142, 10.27) = 8.356, *p* = 0.0136, *n* = 10. * denotes Tukey’s post-hoc comparison of Con (NaCl) vs. KCl, *p* = 0.0215. **(D)** Cortical cultures prepared as per Ding et al. which contain both neurons and astrocytes [see **(A)**] were treated as indicated for 10 min and at 24 h RNA extracted and the SS4^+^/SS4^−^calculated. One way Anova: *F* (1.254, 8.780) = 7.289, *p* = 0.0208, *n* = 8. * denotes Tukey’s post-hoc comparison of Con (NaCl) vs. KCl, *p* = 0.0254.

Analysis of the SS4^+^/SS4^−^ ratio in the RNA from these pure populations revealed that the ratio in neurons is 2 orders of magnitude lower (0.28, Figure 3A) than in astrocytes (36, Figure 3B). Importantly, the KCl treatment protocol of Ding et al. had no impact on the SS4^+^/SS4^−^ ratio in either astrocytes or neurons (Figures 3A,B). This suggests that the authors’ observation of an increased SS4^+^/SS4^−^ ratio in their cultures 24 h after KCl (Ding et al., 2017) is due to neurotoxicity causing an increase in the proportion of astrocytes, rather than an increase in the SS4^+^/SS4^−^ ratio in neurons. To further illustrate this, we mixed RNA samples from pure AF-neuronal cultures and astrocyte cultures, matching the conditions (and taking RNA from equal culture areas), and measured the net SS4^+^/SS4^−^ ratio. There was a significantly higher net SS4^+^/SS4^−^ ratio in the RNA combined from the KCl-treated neurons and KCl-treated astrocytes (Figure 3C), despite the ratio not changing in either cell type individually. The explanation is of course that due to excitotoxicity, there are fewer neurons to extract RNA from in the KCl-treated condition than the other conditions (Figure 2D), so the astrocytic sample makes a greater contribution to the net SS4^+^/SS4^−^ ratio. Note that in our hands, astrocytes express lower total levels of *Nrxn1* than neurons, otherwise the net effect of the increased astrocyte proportion would be even greater. We also studied the effect of KCl on the SS4^+^/SS4^−^ ratio in standard astrocyte-containing neuronal cultures and recapitulate Ding et al.’s observation of an KCl-induced increase in the ratio. However, by overlooking the presence of astrocytes in their cultures, and the possibility of KCl-neurotoxicity, Ding et al. wrongly attribute this increase to an activity-dependent neuronal event. Note also that preventing neuronal death by MK-801 also prevents any change in the SS4^+^/SS4^−^ ratios either when combining RNA samples (Figure 3C) or when analysing the mixed culture (Figure 3D).

The authors did not report neuronal viability data following KCl stimulation, but did publish RNA-seq data of their cultures 24 h after control vs. 10’ KCl. Since we observed a change in the neuron:astrocyte ratio in mixed cultures at this time point, due to death of neurons, we analysed neuron and astrocytic-specific gene expression as a proxy measure of any change in the neuron:astrocyte ratio due to KCl. We generated “neuron-specific” and “astrocyte-specific” gene sets by analysing our own RNA-seq data describing the transcriptome in neuron-free astrocyte cultures and astrocyte-free cortical neuronal cultures (Hasel et al., 2017). Briefly, to define “astrocyte-specific” and “neuron-specific” gene sets, RNA-seq data sets were produced in-house (E-MTAB-8058), sequenced with the Illumina HiSeq 2,500 platform using 50 base pair paired-end reads. RNA was extracted from pure astrocyte cultures and astrocyte-free neuronal cultures (*n* = 3). Genes expressed >10 FPKM and whose expression was 10-fold higher in neurons than astrocytes, or 10-fold higher in astrocytes than neurons (B) were curated.

**Figure 4:**
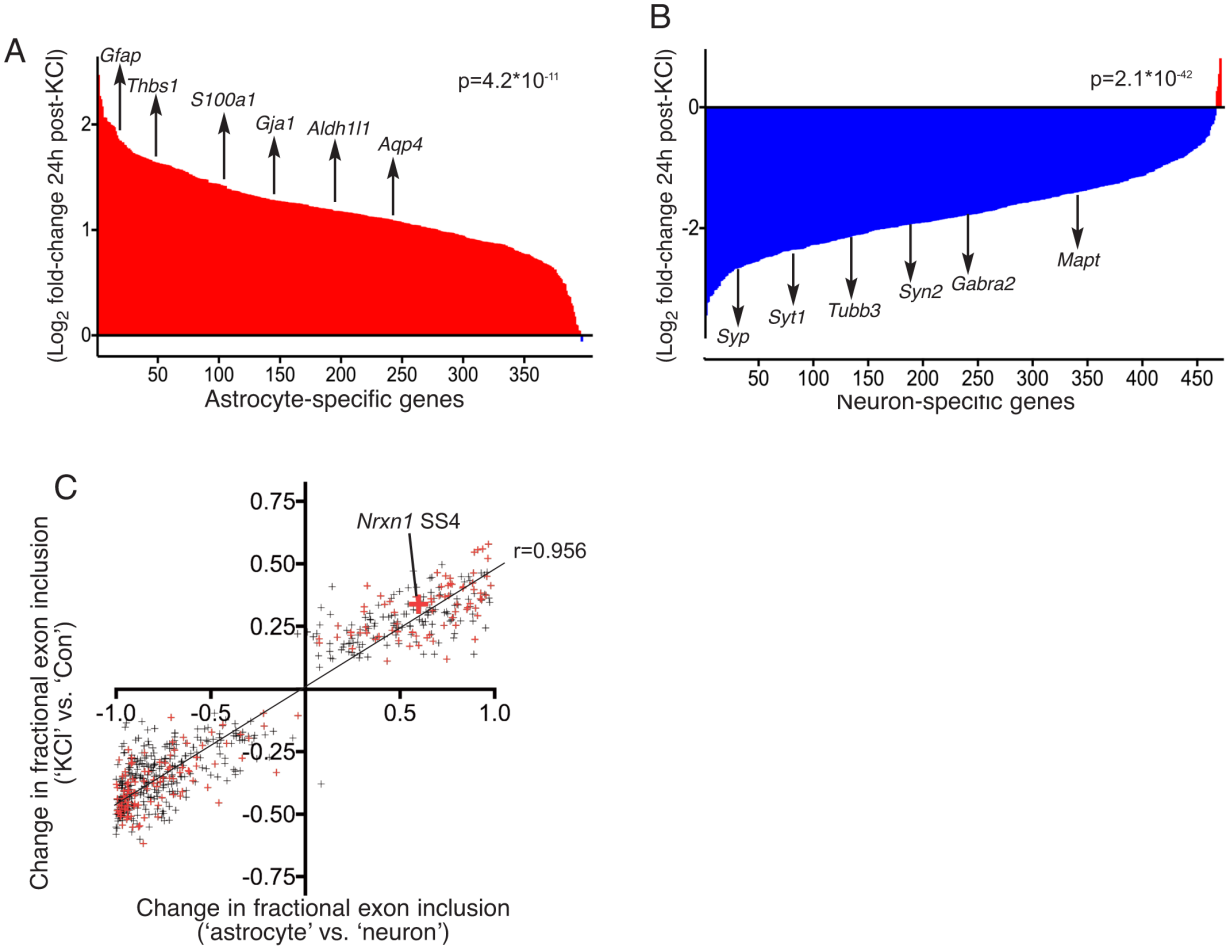
**(A,B)** RNA-seq data sets described by Ding et al. (2017) were downloaded from the GEO database at NCBI, accession GSE93682. Reads in FASTQ format were then mapped to the mouse (mm10) reference genome (using sequence and annotations from Ensembl version 90) with STAR version 2.5.3a. For differential gene expression analysis, reads mapping to genes were counted using featureCounts version 1.5.3, and differential expression was performed using the DESeq2 R package, version 1.16.1. The log(2) fold change of gene expression (KCl vs. Con), was calculated. “Astrocyte-specific” and “neuron-specific” gene sets were curated (see main text for details). For those genes also expressed robustly (>10 FPKM) in the samples of Ding et al., the log(2) fold change of gene expression, comparing (KCl vs. Con) was plotted. For the 470 neuron-specific genes **(A)**, a paired, two-tailed *t*-test was performed on the 470 pairs of FPKM values (Con vs. KCl): *t* = 15.13, df = 469, *p* = 2.06×10^−42^. A selection of neuron-specific genes is highlighted: *Tubb3*, *Mapt, Syp*, *Syt1*, *Syn2* and *Gabra2.* For the 397 astrocyte-specific genes, a paired, two-tailed *t*-test was performed on the 397 pairs of FPKM values (Con vs. KCl): *t* = 6.787, df = 396, *p* = 4.20×10^−11^. A selection of astrocyte-specific genes is highlighted: *Gfap, Aldh1l1, Thbs1, Gja1, S100a1, and Aqp4*. **(C)** The RNA-seq data sets described by Ding et al. (GSE93682, ± KCl) were subjected to differential splicing analysis with rMATS version 3.2.5 (Shen et al., 2014). rMATS was run with a cut-off splicing difference of 0.05 for null hypothesis test for differential splicing (−c option). 612 significantly different levels of exon inclusion (KCl vs. Con) were identified, with a minimum average read-count of 100, and values are plotted on the y-axis as the difference in the fraction of transcripts including the exon (KCl vs. Con). For each of these 612 events, the degree of differential splicing was also calculated between our own neuron-free astrocyte culture and astrocyte-free neuro culture RNA-seq data sets, and plotted on the x-axis. Small red crosses indicate the events identified by Ding et al. in their Supplementary Tables S1 and S2 (Ding et al., 2017). The large red cross highlights the *Nrxn1* SS4 event. Pearson r correlation coefficient = 0.9561, 95% CI: 0.949 to 0.962, *p* < 0.0001. Linear regression slope = 0.4634 (95% CI: 04521 to 0.4747), *p* < 0.0001. Number of X values: 612.

We first sought to ascertain whether the RNA analysed by Ding et al. likely contained a strong contribution from astrocytes in the culture. We compared the expression level of 397 “astrocyte-specific” genes in our pure astrocyte cultures with the “Control” (unstimulated) cultures of Ding et al. (2017). These astrocyte-specific genes were expressed with a FPKM of 30.0 ± 1.4% of that in our pure astrocyte cultures. A level of 30% is indicative that their cultures contain a proportion of astrocytes. We then studied the expression of these 397 astrocyte-specific genes 24 h post-KCl, and found that 396 out of 397 astrocyte-specific genes were enriched 24 h post-KCl (*p* = 4.2 E-11, mean increase: 2.3-fold, Figure 4A). We also looked at the change of 470 “neuron-specific” genes expressed >10 FPKM in the samples of Ding et al., and found that 466 were lower 24 h post-KCl treatment (*p* = 2.06 E-42, mean decrease: 3.4-fold, Figure 4B). These data are consistent with a strong increase in the proportion of astrocytes in their culture 24 h post-KCl and reduction in the proportion of neurons in their culture, aligning with our observations of neurotoxicity of the KCl stimulation (Figures 1A–C).

Ding et al. also reported genome-wide differences in splicing choice 24 h after KCl treatment via the analysis of their RNA-seq data (Ding et al., 2017). We tested the hypothesis that these differences can be simply accounted for by an increase in the proportion of astrocytes in their culture (due to KCl neurotoxicity). If this is the case, the differences observed should align with differences in splicing choice in astrocytes vs. neurons. We analysed their RNA-seq data (KCl vs. Con) and our data (pure astrocytes vs. pure neurons) using rMATS (Shen et al., 2014), the differential splicing analysis tool that Ding et al. employed. We identified 612 distinct differences (including *Nrxn1* SS4) in exon inclusion preference (KCl vs. Con, p_adj < 0.05), and plotted these against the corresponding difference between astrocytes and neurons. We observed a striking positive correlation between differential splicing KCl vs. Con, and differential splicing in astrocytes vs. neurons (*r* = 0.956, 95% CI: 0.949 to 0.962, *p* < 0.0001, Figure 4C). Thus, consistent with an enrichment in astrocyte markers after KCl (Figure 4A), their reported KCl-induced alternative splicing simply reflects the differences in splicing “choices” that astrocytes make compared to neurons.

We conclude that the delayed differentially regulated splicing choices in *Nrxn1* and other genes in neurons reported by Ding et al. are an erroneous conclusion as a result of their stimulation paradigm altering the neuron:astrocyte ratio due to neurotoxicity. Given this, it is hard to rule out the possibility that the reported KCl-induced recruitment of HDAC2 and Suv39h1 to the *Nrxn1* promoter (Ding et al., 2017) also reflects astrocyte vs. neuron differences rather than activity-dependent events. The reported reliance of these processes on AMP-kinase (Ding et al., 2017) could be due to its known role in mediating NMDAR-dependent excitotoxicity (Concannon et al., 2010). While our study was being performed, an independent study (Liakath-Ali and Sudhof, 2021) also investigated the reported activity-dependent alternative splicing of Nrxn1 by Ding et al. (2017). They also found that in mixed cultures of cortical neurons and astrocytes, KCl stimulation (in the absence of NMDAR blockade) kills neurons but not astrocytes, and that this explained the apparent shift in Nrxn1-SS4 alternative splicing due to the SS4^+^:SS4^−^ ratio being higher in astrocytes than neurons. The authors also found that inducing neuronal activity *in vivo* by systemic kainate administration did not lead to *Nrxn1* SS4 alternative splicing in the hippocampus despite clear evidence of enhanced neuronal activity (*Fos* and *Arc* induction), both when analysing whole tissue as well as when analysing neuronally-enriched ribosome-associated mRNA isolated by employing a neuron-specific Ribotag mouse (Liakath-Ali and Sudhof, 2021). Liakath-Ali et al. also observed the strong changes in neuronal (down) and astrocytic (up) gene expression upon KCl stimulation in the RNA-seq data of Ding et al. (2017). To conclude, the influence of activity in controlling alternative splicing in neurons, and the functional consequences of this, is an important topic. However, studies should consider the presence of other cell types in their system, and avoid potentially injurious stimuli.

## Materials and methods

### Cell culture, stimulations and cell death analysis

Neuronal cortical cells were cultured as previously described (Puddifoot et al., 2012) from E17 mouse embryos, at a density of between 9–13 × 10^4^ neurons per cm^2^, and maintained in Neurobasal medium containing 2% B27 supplement, 2 mM glutamine and penicillin/streptomycin for 12 days. These cultures are a mixed population of neurons and astrocytes (85% NeuN+ neurons and 15% GFAP+ astrocytes). To generate highly enriched neuronal cultures (>98% NeuN+ neurons and < 0.2% GFAP+ astrocytes), we followed the same protocol except we added the anti-mitotic drug Cytosine β-D-arabino- furanoside hydrochloride (1.2 mM) immediately post-plating (pure neuronal cultures), as previously described (Bell et al., 2015). Highly enriched astrocyte cultures (>96% GFAP+ astrocytes) were prepared as described (Hasel et al., 2017).

To induce high-potassium mediated membrane depolarization, KCl depolarization buffer (170 mM KCl, 2 mM CaCl_2_, 1 mM MgCl_2_, 10 mM HEPES) was added at 30% final volume, to attain a final K^+^ concentration of 50 mM. After 10 min the cells were returned to regular medium. MK-801 (Tocris) was used where indicated at 10 μM. The control solution was identical in composition to the KCl depolarization buffer except that KCl was replaced by NaCl. Cell death was quantified as previousy described (Baxter et al., 2011). Briefly, neurons were fixed and subjected to nuclear DAPI (Vectorlabs) staining, then imaged using a Leica AF6000 LX imaging system with a DFC350 FX digital camera. Cell death was quantified by counting (blind) the number of pyknotic nuclei as a percentage of the total, with approximately 4,000 cells counted per treatment.

### RNA isolation, RT-PCR and qPCR

RNA was isolated using the Roche High Pure RNA Isolation Kit (including optional DNase treatment), according to manufacturer’s instructions (Roche, Hertfordshire, United Kingdom), with concentrations quantified using a NanoDrop 2000 (Thermo Scientific). For qPCR, cDNA was synthesized from 1–3 μg RNA using the Roche Transcriptor First Strand cDNA Synthesis Kit, according to manufacturer’s instructions. cDNA was then stored at −20°C or immediately diluted (equivalent of 6 ng of initial RNA per 15 μL qPCR reaction, per gene of interest) for real-time PCR in a Stratagene Mx3000P QPCR System (Agilent Technologies, Waldbronn, Germany), using the Roche FS universal SYBR Green MasterRox mix, according to manufacturer’s instructions. The required amount of template was mixed with water, SYBR Green MasterRox mix and forward and reverse primers (200 nM each final concentration) to the required reaction volume. Technical replicates as well as no template and no RT negative controls were included and at least 3 biological replicates were studied per study. Primer sequences are as follows: *Nrxn1*-SS4+ Fwd: CTA CCC TGC AGG AAA CAA TG; Rev.: GCC TCT TCT AGC TGT GCT G (primer pair efficiency 99%); *Nrxn1*-SS4^−^ Fw: CTA CCC TGC AGG GCG; Rev.: GCC TCT TCT AGC TGT GCT G (primer pair efficiency 99%), with underlined nucleotides spanning the respective exon boundaries. Note, in case of any ambiguity regarding *Nrxn1* exon numbering, exon 22 sequence is as follows: GAA ACA ATG ATA ACG AGC GCC TGG CGA TTG CTA GAC AGC GAA TTC CAT ATC GAC TTG GTC GAG TAG TTG ATG AAT GGC TAC TCG ACA AAG. The qRTPCR cycling programme was 10 min. at 95°C; 40 cycles of 30 s. at 95°C, 40 s. at 60°C with detection of fluorescence and 30 s. at 72°C; 1 cycle (for dissociation curve) of 1 min. at 95°C and 30 s. at 55°C with a ramp up to 30 s. at 95°C (ramp rate: 0.2°C/s) with continuous detection of fluorescence on the 55–95°C ramp. Data was analysed using the MxPro qPCR analysis software (Stratagene) with *Nrxn1* isoform expression calculated using the efficiency corrected ΔΔCt method (Livak and Schmittgen, 2001), normalised to *Rpl13a*; Fw: GATGAATACCAACCCCTCC, Rev.: CGAACAACCTTGAGAGCAG (primer pair efficiency 99%).

### Immunofluorescence

This was performed as described previously (McKenzie et al., 2005). Cells were fixed with formaldehyde, washed with phosphate buffered saline (PBS), permeabilised with PBS + NP-40 (Life Technologies Ltd) and washed again prior to overnight rotating incubation with primary antibodies diluted in PBS at 4°C. The following day, cells were washed and antibody binding visualized via biotinylated secondary antibody/fluorophore-conjugated streptavidin. Where required, nuclei were counter-stained with DAPI. In all instances non-saturating images were captured on a Leica AF6000 LX imaging system. Employed primary antibodies include: rat anti-Gfap (1:500, Invitrogen #130300), rabbit anti-NeuN (1:500, Abcam #ab1777487). Fluorescence images were captured on a Leica AF6000 LX imaging system with DFC350 FX digital camera.

### RNA-seq and differential splicing analysis

To generate RNA-seq data, RNA was extracted from astrocyte-free neuronal cultures, and neuron-free astrocyte cultures (see culture methods). Barcoded RNA-seq libraries were prepared by Edinburgh Genomics using the Illumina TruSeq stranded mRNA-seq kit, according to the manufacturer’s protocol (Illumina). The libraries were pooled and sequenced to 50 base paired-end on an Illumina HiSeq 2,500 in high output mode (v4 chemistry) to a depth of approximately 50 million paired-end reads per sample. Reads were mapped to the genomes of each species with version 2.4.0i of the STAR RNA-seq aligner to create output BAM files (Dobin et al., 2013). Subsequently, for each sample, per-gene read counts were summarised using featureCounts version 1.4.6-p2 (Liao et al., 2014). Relative expression levels of genes are expressed as fragments per million reads per kilobase of message (FPKM). Differential splicing events in our data and the published data of Ding et al. were detected using rMATS (Shen et al., 2014), the differential splicing analysis tool that Ding et al. employed. The cut-off criteria for differential exon inclusion choices (Con vs. KCl) was, p_adj < 0.05, average sum of inclusion+exclusion events>100 across the conditions. We plotted this difference against the corresponding difference in exon inclusion choices between astrocytes and neurons, calculated from our own data. Raw data from RNA-seq carried out on RNA extracted from astrocyte-free neuronal cultures, and neuron-free astrocyte cultures is available from European Bioinformatics Institute depository accession reference E-MTAB-8058.

### Statistical analysis and data availability

Paired *t*-tests were used to compare non-independent data pairs, while student *t*-tests were utilized when the two groups were not related. For studies employing multiple testing, we used a one-or two-way ANOVA followed by Tukey’s post-hoc test as appropriate. For all tests, significance was set at *p < 0.05, and all tests were two-tailed. For all experiments the number of replicates (n) used for statistical purposes reflect independent biological replicates of experiments performed on different days involving primary cultures of different tissue origin. Error bars represent standard error of the mean. All data described in the figures is supplied as a Supplementary material (source_data.xlsx). RNA-seq data is available from European Bioinformatics Institute depository accession reference E-MTAB-8058.

## Data availability statement

The datasets presented in this study can be found in online repositories. The names of the repository/repositories and accession number(s) can be found in the article/Supplementary material.

## Ethics statement

The animal study was reviewed and approved by University of Edinburgh Local Animal Ethical Review Board.

## Author contributions

PB performed the experiments. PB, OD, and GH analysed the data. GH conceived the study and wrote the manuscript with edits from PB and OD. All authors contributed to the article and approved the submitted version.

## Funding

This work is supported by the UK Dementia Research Institute and its founding funders the UK Medical Research Council, Alzheimer’s Research UK, and Alzheimer’s Society.

## Conflict of interest

The authors declare that the research was conducted in the absence of any commercial or financial relationships that could be construed as a potential conflict of interest.

## Publisher’s note

All claims expressed in this article are solely those of the authors and do not necessarily represent those of their affiliated organizations, or those of the publisher, the editors and the reviewers. Any product that may be evaluated in this article, or claim that may be made by its manufacturer, is not guaranteed or endorsed by the publisher.

## Supplementary material

The Supplementary material for this article can be found online at: https://www.frontiersin.org/articles/10.3389/fnmol.2023.1214439/full#supplementary-material
